# CXCR4 signaling regulates metastatic onset by controlling neutrophil motility and response to malignant cells

**DOI:** 10.1038/s41598-019-38643-2

**Published:** 2019-02-20

**Authors:** C. Tulotta, C. Stefanescu, Q. Chen, V. Torraca, A. H. Meijer, B. E. Snaar-Jagalska

**Affiliations:** Institute of Biology, Leiden University, Gorlaeus Laboratories, Einsteinweg 55, 2333 CC Leiden, The Netherlands

## Abstract

Developing tumors interact with the surrounding microenvironment. Myeloid cells exert both anti- and pro-tumor functions and chemokines are known to drive immune cell migration towards cancer cells. It is documented that CXCR4 signaling supports tumor metastasis formation in tissues where CXCL12, its cognate ligand, is abundant. On the other hand, the role of the neutrophilic CXCR4 signaling in driving cancer invasion and metastasis formation is poorly understood. Here, we use the zebrafish xenotransplantation model to study the role of CXCR4 signaling in driving the interaction between invasive human tumor cells and host neutrophils, supporting early metastasis formation. We found that zebrafish *cxcr4 (cxcr4b)* is highly expressed in neutrophils and experimental micrometastases fail to form in mutant larvae lacking a functional Cxcr4b. We demonstrated that Cxcr4b controls neutrophil number and motility and showed that Cxcr4b transcriptomic signature relates to motility and adhesion regulation in neutrophils in tumor-naïve larvae. Finally, Cxcr4b deficient neutrophils failed to interact with cancer cells initiating early metastatic events. In conclusion, we propose that CXCR4 signaling supports the interaction between tumor cells and host neutrophils in developing tumor metastases. Therefore, targeting CXCR4 on tumor cells and neutrophils could serve as a double bladed razor to limit cancer progression.

## Introduction

Tumor-microenvironment interactions are crucial in cancer pathogenesis and several signals drive this communication^1^. The composition of cancer microenvironments changes during cancer progression^2^. Fibroblasts, endothelial and immune cells are main components of the tumor stroma, acting in concert with the extracellular matrix (ECM), growth factors, proteases and cytokines^3^. The CXCR4-CXCL12 chemokine signaling axis sustains tumor cell growth and directs the formation of distant metastases. It is established that cancer cells expressing CXCR4 home to secondary organs where CXCL12 is highly secreted, mainly by mesenchymal stromal cells^4^. Moreover, CXCL12 guides the migration of stromal cells that express CXCR4 and locally infiltrate the tumor, providing support by secretion of growth and angiogenic factors, as well as promoting metastasis through activation of epithelial-to-mesenchymal transition (EMT) via mitogen-activated protein kinase (MAPK), phosphoinositide 3-kinase/Protein kinase B (PI3K/AKT) and nuclear factor kappa-light-chain-enhancer of activated B cells (NFKB) pathways^3,5^.

A dual role in either supporting or inhibiting tumor progression has been linked with the immune system^1^. CXCR4-CXCL12 signaling has been associated with the polarization towards an immune-suppressive microenvironment: the possible role of a CXCL12 shield that protects cancer cells from being recognized by cytotoxic lymphocytes and activates regulatory T-cells has recently been described^6^. Polarization of macrophages towards a M2 phenotype has also been associated with tumor survival. Recent studies have pointed at the role of perivascular CXCR4-expressing M2 macrophages in creating tumor vascular networks after chemotherapy, leading to tumor relapse, and confirmed CXCR4 as M2 marker^7^. It has been shown that CXCR4 can also be activated by alternative ligands like MIF (Macrophage Migration Inhibitory Factor)^8^. MIF signalling has been associated to inflammatory diseases. Upon binding to CXCR4 or CXCR2, MIF controls monocyte and T cell chemotaxis and its blockade leads to plaque regression in atherosclerosis^8^. In zebrafish, MIF functions as a neurotrophin during the development of the inner ear^9^. In cancer, MIF-CXCR4 signalling has been linked to Mesenchymal Stromal Cell (MSC) homing to tumours both *in vitro* and *in vivo*^10^. The FDA-approved CXCR4 antagonist AMD3100 inhibits MIF binding to CXCR4. However, because higher concentration of the antagonist is required to inhibit MIF binding to CXCR4 compared to CXCL12, it is likely that MIF binds to CXCR4 via a different mechanism compared to CXCL12 binding^11^. We previously showed that metastasis formation is inhibited in a cxcl12 zebrafish mutant, suggesting a pivotal role of the cxcl12-cxcr4 signaling axis in this process^12^.

Neutrophils are the most abundant white blood cells and the major first responders during inflammation^13^. In cancer, neutrophils are recruited to neoplastic sites and together with other immune cells have been shown to provide trophic signals that support tumor growth, angiogenesis, tumor cell motility and invasion of surrounding tissues^14–17^. Neutrophils have been classified in N1 (anti-tumor) and N2 (pro-tumor) types^18–20^. The polarization of neutrophils towards one or the other type is driven by a plethora of cytokines and chemokines that often direct the same polarization in macrophages. In particular, pro-inflammatory molecules such as interferon β (IFNβ), interleukin-1β (IL1β) and tumour necrosis factor α (TNFα) induce the polarization towards type 1 phenotypes, while interleukin 10 (IL-10) and transforming growth factor β (TGFβ) are immunosuppressive and inhibitory of inflammation, skewing neutrophil polarity towards N2. Pro-tumoral and pro-angiogenic N2 neutrophils express high levels of vascular endothelial growth factor (VEGF), metalloprotease 9 MMP9 and CXCR4^20^. In addition, amongst different metalloproteases, MMP9 plays a key role in HSCs mobilisation from the bone marrow. CXCR4 expression is regulated by MMP9. Simultaneously MMP9 and CXCL12 expression is reciprocally regulated in bone marrow cells^21^.

Neutrophils have been reported to display overlapping as well as complementary functions with macrophages in infection and tumor relapse after chemotherapy^22,23^. Interestingly, tissue-resident macrophages, originated from the fetal liver during embryo development, and monocyte-derived macrophages, originated from hematopoietic stem and progenitor cells (HSPCs) in the adult bone marrow, work in concert to regulate recruitment of neutrophils in inflamed tissues, through epithelial layers^24^. Recent findings suggest that neutrophils work together with macrophages to regulate the hematopoietic niche^25^. The bone microenvironment represent a favorable site of metastatic growth for different tumor types, suggesting a possible involvement of the signals that regulate bone marrow and hematopoietic niche homeostasis^26^. Among those, CXCR4-CXCL12 signaling is a major candidate, considering its fundamental role in orchestrating HSPC and neutrophil retention in and mobilization from the bone marrow, with the involvement of the CXCL1/CXCL2-CXCR2 chemokine axis^27–29^.

The use of the zebrafish embryo as a xenotransplantation model has shown that hematogenously inoculated tumor cells home in the caudal hematopoietic tissue (CHT), where tumor growth and invasion take place, initiating early metastatic events^30^. The CHT is an intermediate site of hematopoiesis during zebrafish embryogenesis and is the functional analogue of the fetal liver in mammalian development^31^. Previous work from our group has suggested the role of neutrophils in preparing the metastatic niche by non-pathological transmigration from the CHT to the tail fin and *vice versa*. In their random motility, neutrophils form paths in the collagen, favoring tumor cell invasion^30^. We previously addressed the role of cell-autonomous CXCR4 signaling in early metastases in the zebrafish xenograft model^12^. Here, we address the role of the host-dependent CXCR4 signaling in driving the communication between tumor cells and neutrophils, during experimental metastasis formation in an *in vivo* zebrafish xenogeneic model.

## Results

### Myeloid cells support tumor early metastatic events

**Figure 1:**
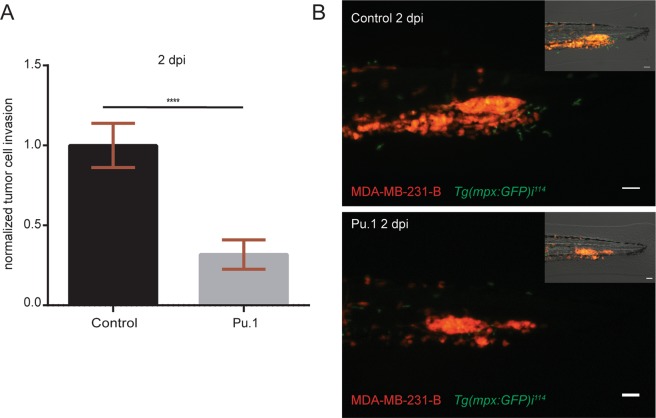
Myeloid cell depletion impairs tumor cell invasion. (**A**) Relative tumor invasion was compared at 2 dpi in Pu.1 morphants, depleted of neutrophils and macrophages, and larvae injected with control morpholino (68% inhibition). Two-tailed un-paired t-test with Welch’s correction (****p < 0.0001) was performed on a pool of two biological replicates (Control: n = 84, Pu.1: n = 67). Data are mean ± SEM. (**B**) Top panel shows MDA-MB-231-B cells forming a tumor mass and invading the tail fin tissue (bright field image, top right), while surrounded by GFP expressing neutrophils in 2 dpi *Tg(mpx:GFP)*^*i114*^ injected with a control morpholino. In the bottom image, neutrophils are absent due to Pu.1 knockdown and a smaller tumor mass is formed compared to the control condition, resulting in impaired invasion of the local tissue (bright field, top right). Scale bar: 50 µm. Micrographs were acquired using a Leica MZ16FA fluorescent microscope coupled to a DFC420C camera.

Immune cells play dual roles during cancer progression. Inhibitory and supportive functions of the immune system have been associated with tumor growth and metastasis formation. Using the zebrafish embryo model we previously showed that myeloid cells, mainly neutrophils, support the establishment of tumor experimental micrometastasis, when the MAE-FGF2 transformed cell line was inoculated into the blood circulation of 2-day-old embryos^30^. Therefore, we used the same approach to investigate whether zebrafish myeloid cells exert similar tumor supportive functions, when other cell lines were implanted. In particular, we used the osteotropic triple negative breast cancer line MDA-MB-231-B, derived from bone metastases in a mouse xenograft model^32^. The zebrafish embryo model bears the great advantage of studying the contribution of the innate immune system during early metastasis formation separately from the adaptive immunity, which reaches full maturity in 3–4 week old juveniles^33^. To deplete both neutrophils and macrophages, we injected Pu.1/Spi1b morpholino (1 mM) into 1–2 cell stage embryos. Subsequently, the MDA-MB-231-B cell line was inoculated into the blood circulation of 2-day post fertilization (dpf) zebrafish embryos with GFP-expressing neutrophils. The reporter line *Tg(mpx:GFP)*^*i114*^ ^34^ was used to monitor neutrophil depletion, in view of the time-limited efficacy of gene knock-down obtained with morpholino anti-sense oligos. Macrophage depletion was not monitored as it already occurs with lower doses of the same morpholino (0.5 mM)^30^. Tumor phenotype assessment was performed 2-day post implantation (dpi) by quantifying tumor cell invasion in each larva. Depletion of myeloid cells in the Pu.1 morphants resulted in a reduced cancer cell invasion (68%) in the tail fin in proximity of the caudal hematopoietic tissue (CHT) (Fig. 1). As previously found, the CHT, a site of hematopoiesis and analogous to the fetal liver during mammalian development, is a preferential site of early cancer metastasis formation in the zebrafish xenotransplantation model. In conclusion, myeloid cells support triple negative breast cancer early metastasis onset in zebrafish.

### Neutrophilic Cxcr4 signaling is involved in early tumor metastasis initiation

Therapeutic targeting of CXCR4 on tumor cells could be an effective strategy to limit tumor cell growth and metastasis. However, CXCR4 signaling in the tumor microenvironment also plays a central role in cancer and further investigations are needed to fully understand its contribution.

**Figure 2:**
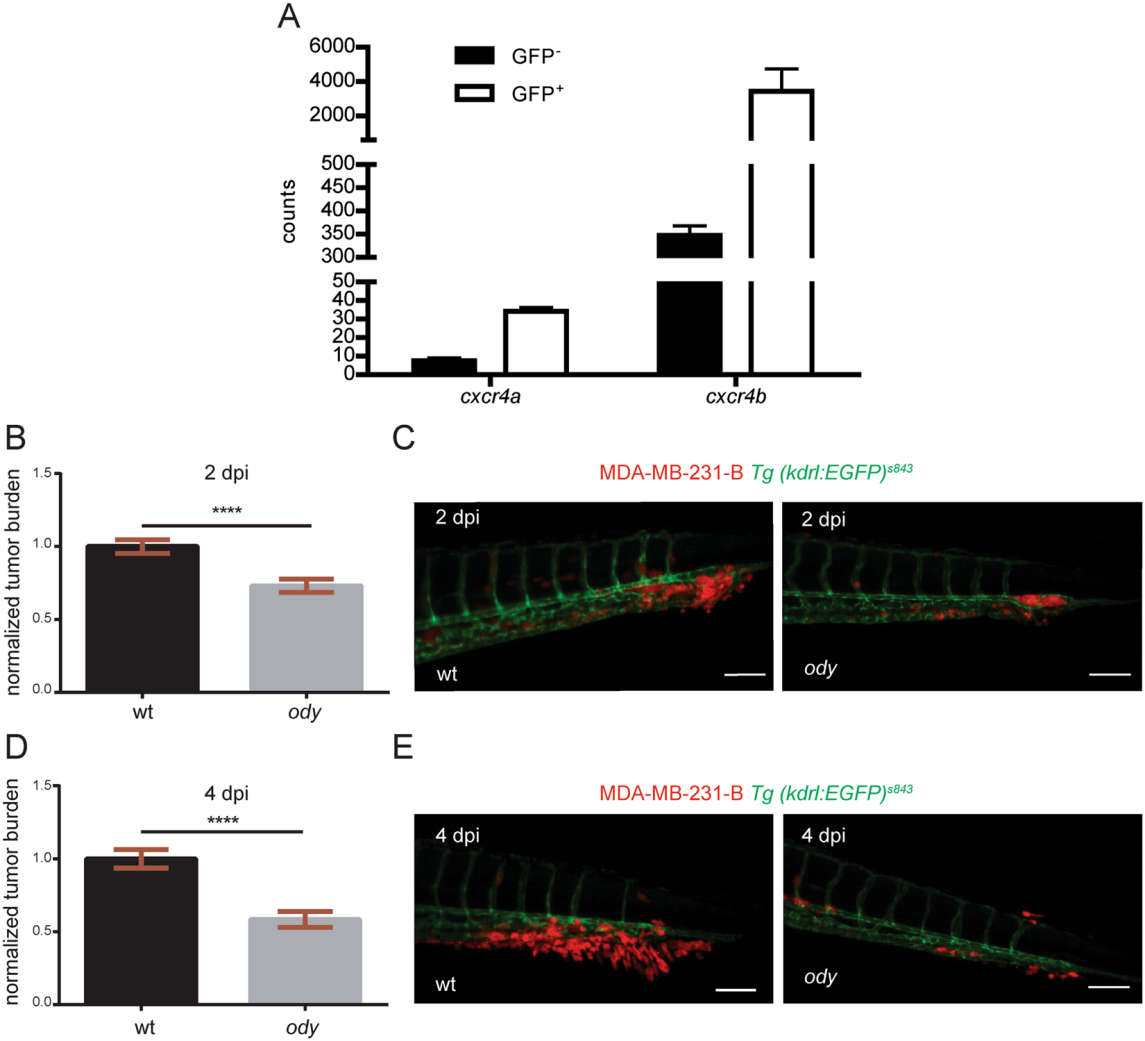
c*xcr4b* is highly expressed in neutrophils and loss of function results in reduced triple negative breast cancer burden. (**A**) *cxcr4a* and *cxcr4b* expression levels were quantified in neutrophils and compared to the GFP negative cell population. Data are read counts from RNA sequencing performed on three biological replicates. FACS-sorted neutrophils were obtained from 5 dpf *Tg(mpx:GFP)*^*i114*^ larvae. *cxcr4a* and *cxcr4b* gene expression was enriched in neutrophils compared to GFP negative cells in zebrafish larvae (~4-fold and ~10-fold, respectively). *cxcr4b* was highly expressed in neutrophils compared to *cxcr4a* (~100-fold increased gene expression). (**B**) Relative metastatic tumor burden of MDA-MB-231-B-DsRed cells was quantified in *ody* and wt siblings at 2 dpi. Data are mean ± SEM of two independent experiments (wt: n = 64, *ody*: n = 57). Un-paired t-test ****p < 0.0001. (**C**) MDA-MB-231-B tumor cells established a secondary tumor mass, with initiation of single cell extravasation, in wt larvae, whereas a phenotype inhibition was found in *ody* mutants at 2 dpi (22.5% reduction). (**D**) MDA-MB-231-B tumor burden was measured in wt and *cxcr4b* null mutants at 4 dpi, at the metastatic site where secondary growth began at 2 dpi. A 40.5% reduction in tumor burden was observed. Data are mean ± SEM of two independent experiments (wt: n = 59, *ody*: n = 43). Un-paired t-test, with Welch’s correction ****p < 0.0001. (**E**) Highly invasive cancer cells displayed aggressive and metastatic features in wt siblings, whereas few cells remained in the CHT region of 4 dpi *ody* larvae. Scale bars: 50 µm. Micrographs are acquired using a Leica MZ16FA fluorescent microscope coupled to a DFC420C camera.

**Figure 3:**
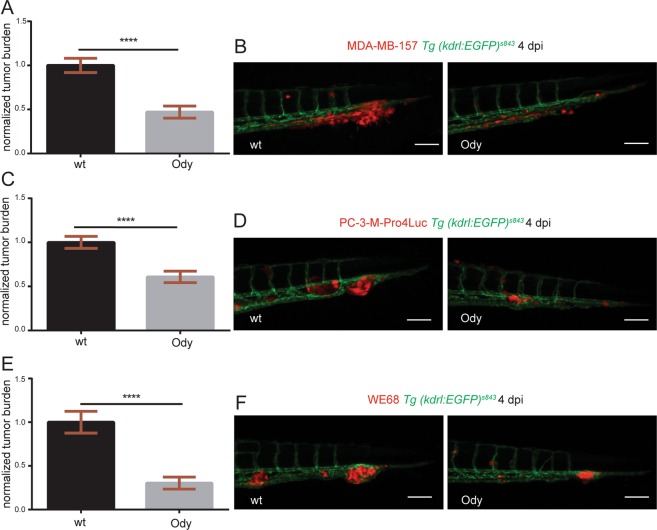
c*xcr4b* deficient host blocks metastatic burden of different tumor types. (**A**) Metastatic burden was assessed in 4 dpi zebrafish larvae engrafted with the triple negative breast line MDA-MB-157 mCherry. A 52% reduction was found. Data are mean ± SEM of two independent experiments (wt: n = 42, *ody*: n = 28). Un-paired t-test, with Welch’s correction ****p < 0.0001. (**B**) Secondary tumor mass, extravasation and invasion failed to occur in *ody* mutants compared to wt siblings. (**C**) A significantly lower tumor burden in *cxcr4b* deficient larvae was observed when the prostate cancer PC3-M-Pro4-Luc2 mCherry or td-tomato cell line was implanted (38% reduction). Data are mean ± SEM of two independent experiments (wt: n = 48, *ody*: n = 46). Un-paired t-test ****p < 0.0001. (**D**) Prostate cancer early metastasis formation, characterized by a solid tumor mass formation in the CHT region of zebrafish larvae, occurred in wt siblings and was significantly decreased when Cxcr4b signaling was impaired in the host. (**E**) Relative metastatic burden of Ewing sarcoma cell line WE-68 td-tomato was affected in *ody* mutants compared to wt larvae at 4 dpi (70% reduction in tumor burden in the tail fin). Data are mean ± SEM of two independent experiments (wt: n = 69, *ody*: n = 39). Un-paired t-test, with Welch’s correction ****p < 0.0001. (**F**) Ewing sarcoma cells formed a compact and expanding tumor mass in the CHT region, between the dorsal aorta and the caudal vein. A reduced tumor cell aggregate was present in the *ody* mutant line at 4 dpi. Scale bars: 50 µm. Micrographs were acquired using a Leica MZ16FA fluorescent microscope coupled to a DFC420C camera.

**Figure 4:**
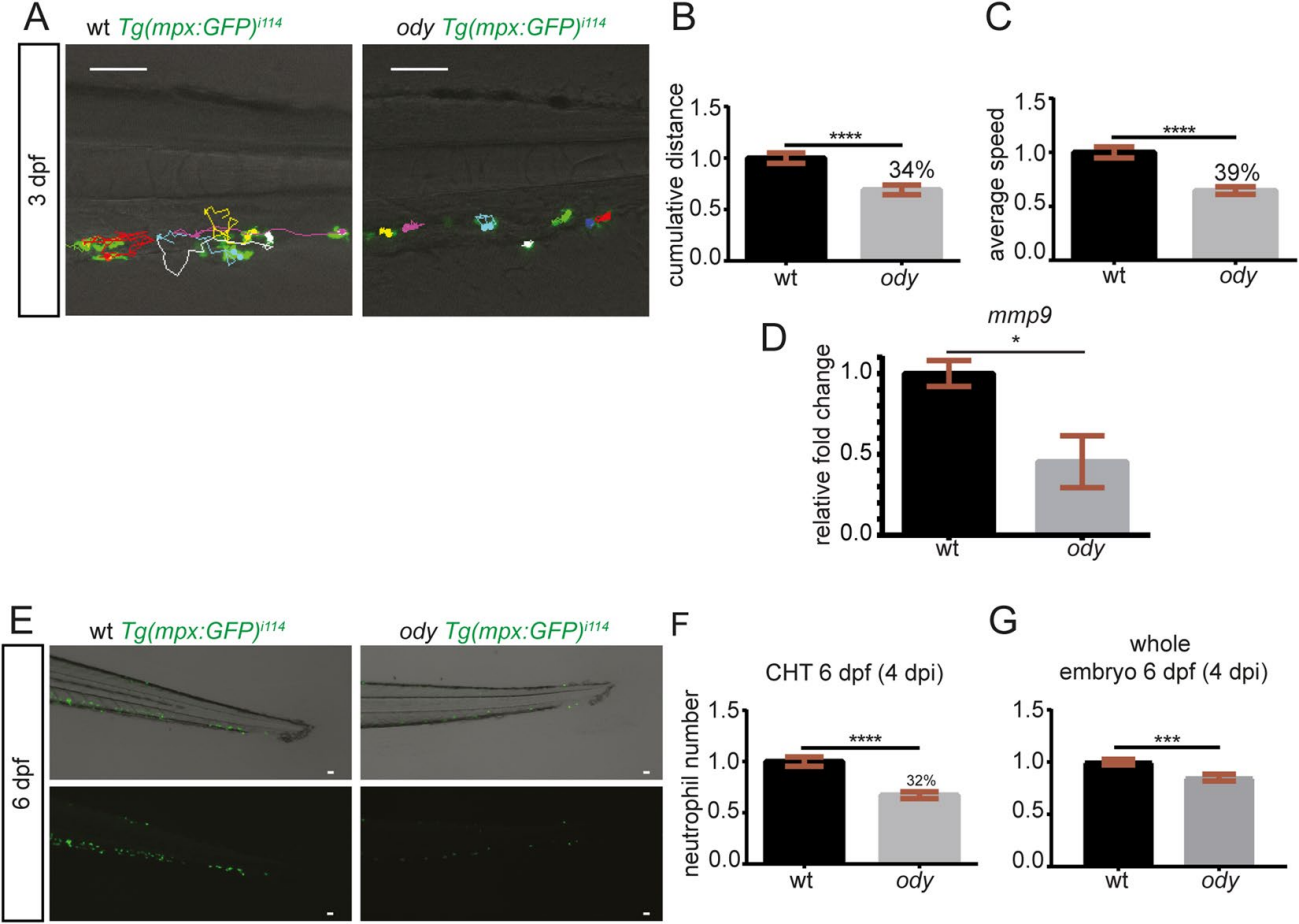
c*xcr4b* deficiency affects neutrophil physiological motility and development. (**A**) Neutrophil movement was recorded for 30 minutes and tracks showed reduced motility in *ody* compared to wt siblings in the tail fin region where tumor metastasis formation generally takes place. Scale bars: 50 µm. Time-lapse microscopy was performed using a Leica TCS SPE confocal microscope with a HC APO 20x DRY objective (0.7 N.A.). Neutrophil motility was assessed in wt and *ody* larvae at 3 dpf, measuring cumulative distance (**B**) and average speed (**C**) of each phagocyte, localized in the CHT region. (**B**) Un-paired t-test **** p < 0.0001 and (**C**) Un-paired t-test, with Welch’s correction ****p < 0.0001. Data are mean ± SEM of two independent experiments and values were calculated from 54 tracks (wt: n = 7) and 58 tracks (*ody*: n = 8). (**D**) mmp9 expression in 6 dpf *ody* and wt siblings. *p = 0.02, unpaired t-test. (**E**,**F**) Number of neutrophils in wt and *ody* in the CHT region at 6 dpf is shown. A lower neutrophil number was found in the CHT region in *cxcr4b* ^−/−^ larvae (32% reduction), as shown by top and bottom micrographs (**E**) and quantified in (**F**). Un-paired t-test **** p < 0.0001. Data are mean ± SEM of two independent experiments (wt: n = 35, *ody*: n = 36). (**G**) A significant reduction in total neutrophil number was found in *ody* larvae at 6 dpf. In (**G**) wt: n = 35, *ody*: n = 36. Data are mean ± SEM (pool of two independent experiments). Un-paired t-test with Welch’s correction ***p = 0.0007.

In our model, teleost evolution has led to a *cxcr4* gene duplication. *cxcr4a* and *cxcr4b* paralogues are expressed by different cell types, although redundant functions have been reported^35^. The CXCL12-CXCR4 signaling is conserved between zebrafish and human: zebrafish Cxcr4 receptors display more than 60% identity with human CXCR4 and zebrafish Cxcl12 ligands have more than 65% identity with human CXCL12^12^. We performed transcriptome analysis of GFP positive, FACS-sorted neutrophils from 5 dpf *Tg(mpx:GFP)*^*i114*^ larvae and RNA deep sequencing revealed high expression levels of the *cxcr4* paralogues in neutrophils. In particular, *cxcr4a* and *cxcr4b* transcriptomic levels were higher in the GFP^+^ fractions compared to the GFP^−^ populations. Importantly, neutrophilic *cxcr4b* levels were at least 100-fold higher than neutrophilic *cxcr4a*, indicating that *cxcr4b* is the predominant human *CXCR4* orthologue in zebrafish larval neutrophils (Fig. 2A). Therefore, to study if CXCR4 signaling in the tumor microenvironment supports cancer metastasis initiation, we engrafted the triple negative breast cancer cell line MDA-MB-231-B in the *cxcr4b*^*t26035*^ (*odysseus* or o*dy*) mutant with deficient *cxcr4b*^36^. Xenogeneic transplantation into the blood circulation via the duct of Cuvier resulted in a strong proliferating and invasive tumor phenotype, characterized by experimental micrometastasis formation in the CHT region in the wild-type (wt) siblings, whereas a significant reduction was observed in the *cxcr4b*^*−/−*^, *ody* mutants (Fig. 2B–E). The establishment of early metastatic events defined by tumor mass formation and extravasation followed by local tissue invasion was monitored by fluorescence at 2 (Fig. 2B,C) and 4 (Fig. 2D,E) days after engraftment and tumor burden was found to be significantly inhibited in *ody* larvae (22.5% and 40.5% reduction at 2 and 4 dpi, respectively). In order to test whether Cxcr4 signaling inhibition in the microenvironment could affect the metastatic cascade in other tumor types, we engrafted another triple negative breast cancer cell line MDA-MB-157 (Fig. 3A,B), as well as prostate cancer cells PC3-M-Pro4-Luc2 (Fig. 3C,D) and the Ewing sarcoma cell line WE68 (Fig. 3E,F). Tumor early metastasis establishment in the CHT region of 4 dpi zebrafish larvae was impaired in the *ody* mutant line compared to the wt siblings, when each cell line was inoculated into the blood circulation (reduction of tumor burden was 52%, 38% and 70% in breast, prostate and Ewing sarcoma tumor cell lines, respectively) (Fig. 3). Therefore, we suggest that neutrophilic Cxcr4 signaling plays a crucial role in the early steps of metastases formation of triple negative breast cancer as well as other tumor types.

### Cxcr4b signaling inhibition attenuates neutrophil basal motility and development

CXCR4 signaling has been found to play an important role in regulating neutrophil retention in the CHT in the WHIM syndrome, where neutropenia has been linked to increased susceptibility to infection in patients as well as in the zebrafish model^37,38^. Therefore, as neutrophils express high levels of *cxcr4b*, we investigated whether the impairment of Cxcr4 signaling affects the motility of neutrophils in the CHT region, altering their ability to prepare the metastatic niche. Neutrophil migration under physiological conditions was recorded for 30 min as previously described^30^. Neutrophils displayed reduced motility when Cxcr4b signaling was impaired in the *ody* mutant compared to the wt siblings (Fig. 4A–C). We have previously shown that neutrophils prepare the metastatic niche by creating paths into the collagen, during the transmigration from the CHT to the tail fin. Hence, we hypothesized that path formation is linked to metalloprotease activity. Therefore, we quantified mmp9 expression in *ody* and wt siblings (whole body) and found decreased mRNA levels upon Cxcr4b inhibition (Fig. 4D).

Next, neutrophil number in the CHT and whole body was verified at 6 days post fertilization when metastasis formation was assessed. Neutrophil number was lower in the CHT of *ody* mutants compared to wt siblings (Fig. 4E,F). Moreover, at the same time point, the total body count of neutrophils was found to be lower (Fig. 4G), suggesting that Cxcr4b controls neutrophil development.

During zebrafish development, primitive and definitive waves of hematopoiesis can be distinguished. In a transition phase, between 24 and 36 hpf, neutrophils originate from the posterior blood island (PBI), which, with the onset of the definitive wave, is replaced by the CHT^39^. Recent studies in zebrafish have revealed that CXCR4 signaling has a direct link with the development of HSPCs, mainly affecting their ability to colonize the CHT, which functions as an intermediate hematopoietic site^40^. In the same study, the use of the CXCR4 antagonist AMD3100, between 48 and 72 hpf, decreased *cmyb/runx*^+^ HSPCs numbers. Because neutrophils develop first in the PBI, independently from the HSPCs, and subsequently in the CHT, dependently on the HSPCs with self-renewal potential, we investigated whether the development of neutrophils could be affected in a host with a non-functional Cxcr4b signaling. Neutrophil number was quantified during earlier stages of development (1 dpf), before HSPCs colonize the CHT and initiate the definitive wave of hematopoiesis. An increase in neutrophil number was found in the CHT of *ody* embryos, compared to wt siblings, whereas no difference was detected on whole embryo level (Fig. S1A–C). Subsequently, neutrophil number was quantified in the whole zebrafish embryo, as well as in the CHT region, in between the dorsal aorta and caudal vein, starting from the end of the yolk extension, in 2 day old *cxcr4b*^*−/−*^ and *cxcr4b*^+*/*+^ *Tg(mpx:GFP)*^*i114*^ embryos. We identified an increase (31%) in neutrophil number in the CHT region of *ody* mutants compared to wt siblings (Fig. S1D,E) at 2 dpf. At the same time, no difference in total neutrophil number was observed (Fig. S1F).

These findings suggest that Cxcr4b controls neutrophil motility and development, in a putative HSPCs-dependent and independent manner.

### The transcriptomic signature of Cxcr4b-deficient neutrophils links to defective cell motility

**Figure 5:**
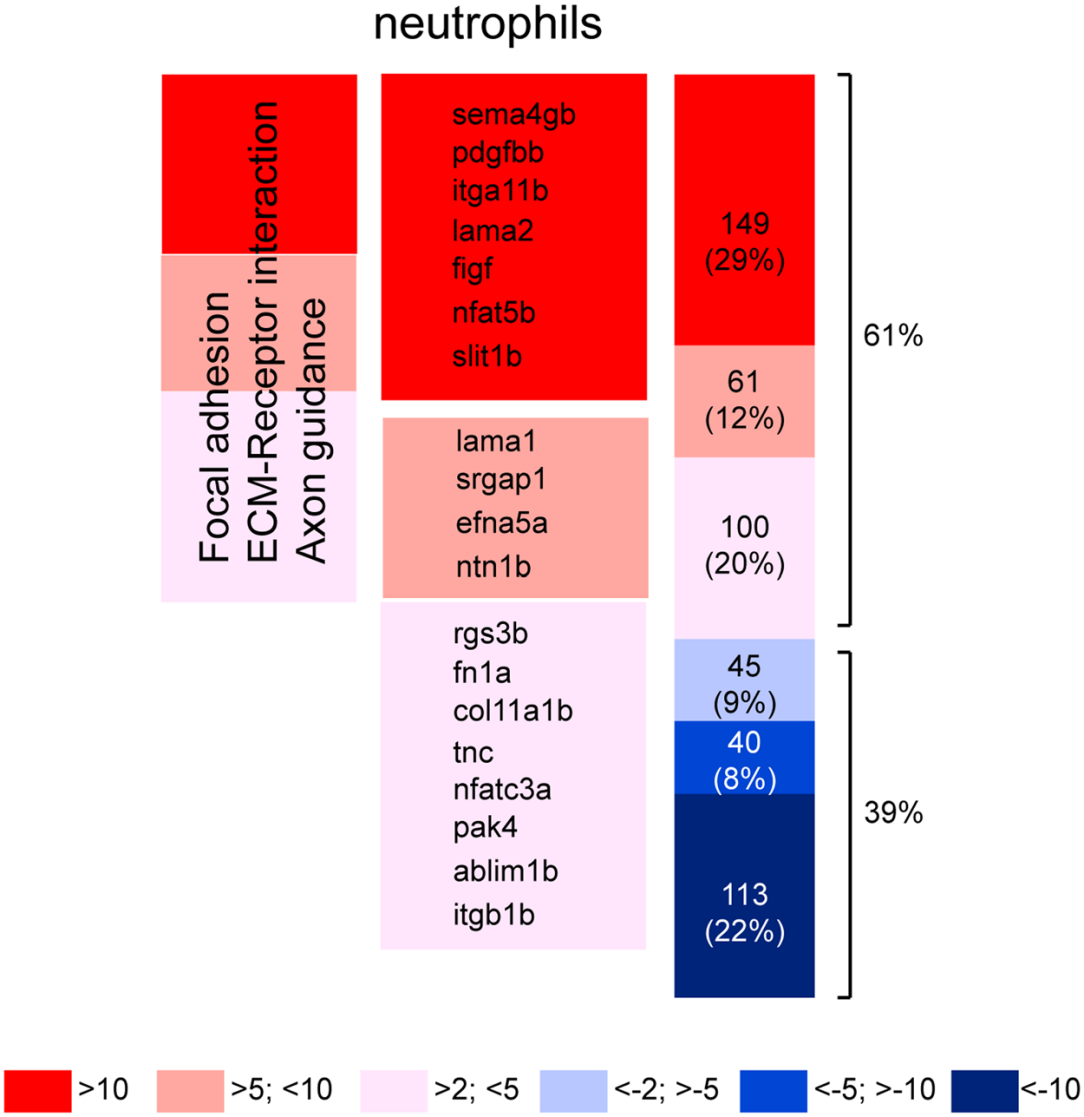
Cxcr4b transcriptomic signature in zebrafish neutrophils. (**A**) Heatmap showing up- and down-regulated genes in *cxcr4b*^−/−^ neutrophils compared to *cxcr4b*^+/+^ neutrophils. 61% is the percentage of up-regulated genes, whereas 39% is the percentage of down-regulated genes. Genes involved in focal adhesion, ECM-Receptor interaction and axon guidance are up-regulated. Percentages of up- or down-regulation are calculated based on the total number of genes left after a cutoff of p < 0.05 in both DESeq and edgeR (neutrophil dataset: from n = 21517 to n = 508). Before the analysis, 107 genes were manually removed in the neutrophil dataset due to high variation among the triplicates. An alternative analysis method using DESeq2 paired confirmed the affected pathways (Table 2).

In this study (Fig. 4A–C) we demonstrated that neutrophil motility is altered in physiological conditions when Cxcr4b signaling is impaired. In order to define the contribution of neutrophilic Cxcr4b signaling axis involved in metastatic niche preparation and subsequent tumor cell invasion, RNA sequencing was performed from FACS-sorted GFP positive neutrophils after dissociation of cxcr4b^+/+^ and cxcr4b^−/−^ *Tg (mpx:GFP)*^*i144*^ 6 dpf larvae. An overall tendency towards upregulation of differentially expressed genes was found in neutrophils from *ody* mutants (61% upregulated genes vs 39% downregulated genes) (Fig. 5), when a cutoff was considered (p < 0.05 in DESeq and edgeR). More in details, in *ody* neutrophils 48% of the up-regulated genes showed an over 10-fold increase, whereas 57% of the down-regulated genes showed an over 10-fold decrease. Pathway analysis was performed in DAVID, after selecting 615 differentially expressed genes (p < 0.05 in DESeq and edgeR) and converting them to human orthologues with gPROFILER. Genes involved in focal adhesion and ECM-receptor interaction were found up-regulated in neutrophils, together with genes involved in axon guidance, suggesting impaired motility and anchoring properties (Table 1). In particular, integrins are involved in adhesion strengthening and arrest of leukocytes on the endothelium, during transendothelial migration^41^. Laminin, fibronectin and collagen are components of the extracellular matrix and increased transcription levels suggest a tighter adhesion ad consequently challenged immune cell motility (Table 1). Members of the Roundabout signaling pathway (*slit1b*, *sema4gb* and *srgap1*), implicated among others in leukocyte chemotaxis and tumor angiogenesis^42^ were found to be up-regulated. Down-regulated genes were found to cluster in the metabolism of xenobiotics by cytochrome p450 pathway. Subsequently, pathway analysis was extended to differentially expressed genes identified through statistical analysis performed in RStudio using the package DESeq2 paired. Overall, the analysis performed in DESeq2 paired confirmed the enriched pathways identified with DESeq and edgeR. However, additional genes were identified, either belonging to previously described pathways (focal adhesion/ECM-Receptor interaction) or clustering in a new pathway (MAPK pathway) (Table 2). Furthermore, *NETRIN-1* (zebrafish *netrin1b*), belonging to the family of laminin-secreted proteins and involved in neuronal chemotaxis^43,44^ and leukocyte migration^45^, was found up-regulated in *cxcr4*^−/−^ neutrophils (Log_2_FoldChange = 2.6 and p = 0.00009). *NETRIN-1* has previously been linked with reduced neutrophil and macrophage infiltration in a kidney injury model^46^. Taken together, our sequencing data support the above described results that suggest motility alteration in neutrophils bearing a *cxcr4b* mutation.

**Table 1:** Enriched pathways in *cxcr4b* ^−/−^ neutrophils (analysis performed with DESeq and edgeR).

			DESEq		edgeR	
Gene ID	Gene symbol	Gene name	LogFC	p	LogFC	p
**Focal adhesion/ECM-receptor interaction**						
ENSDARG00000056624	figf	c-fos induced growth factor	3.9	3.0E-02	3.9	3.0E-02
ENSDARG00000009014	col11a1b	collagen, type XI, alpha 1b	1.8	6.0E-03	1.8	5.0E-03
ENSDARG00000019815	fn1a	fibronectin 1a	1.9	3.0E-02	1.9	3.0E-02
ENSDARG00000007950	itga11b	integrin, alpha 11b	4.8	3.0E-03	4.6	3.0E-03
ENSDARG00000053232	itgb1b.1	integrin beta 1b.1	1.2	4.0E-02	1.2	4.0E-02
ENSDARG00000102277	lama1	laminin, alpha 1	3.1	9.0E-03	3.1	1.0E-02
ENSDARG00000099390	lama2	laminin, alpha 2	4	1.0E-02	3.9	2.0E-02
ENSDARG00000018110	pak4	p21 protein (Cdc42/Rac)-activated kinase 4	1.5	2.0E-02	1.5	1.0E-02
ENSDARG00000038139	pdgfbb	platelet-derived growth factor beta polypeptide b	6.8	8.0E-04	6.3	6.0E-03
ENSDARG00000078362	tnc	tenascin C	1.6	2.0E-02	1.6	3.0E-02
**Axon guidance**						
ENSDARG00000007461	srgap1	SLIT-ROBO Rho GTPase activating protein 1	2.9	4.0E-02	2.9	4.0E-02
ENSDARG00000045064	ablim1b	actin binding LIM protein 1b	1.3	2.0E-02	1.3	2.0E-02
ENSDARG00000089790	efna5a	ephrin-A5a	2.8	1.0E-02	2.8	3.0E-02
ENSDARG00000053232	itgb1b.1	integrin beta 1b.1	1.2	4.0E-02	1.2	4.0E-02
ENSDARG00000022531	ntn1b	netrin 1b	2.6	9.0E-05	2.6	7.0E-03
ENSDARG00000102556	nfat5b	nuclear factor of activated T-cells 5	3.6	1.0E-02	3.5	2.6E-02
ENSDARG00000076297	nfatc3a	nuclear factor of activated T-cells, cytoplasmic 3a	1.6	2.6E-02	1.6	3.2E-02
ENSDARG00000018110	pak4	p21 protein (Cdc42/Rac)-activated kinase 4	1.5	2.2E-02	1.5	1.1E-02
ENSDARG00000035132	rgs3b	regulator of G-protein signaling 3b	2.3	3.0E-03	2.3	5.0E-03
ENSDARG00000088143	sema4gb	semaphorin 4gb	1.00E + 06	5.9E-04	7.9	4.5E-04
ENSDARG00000099446	slit1b	slit homolog 1b	3.5	6.6E-03	3.5	2.3E-02
**Metabolism of xenobiotics by cytochrome P450**						
ENSDARG00000006220	ugt1ab	UDP glucuronosyltransferase 1 family a, b	−2.6	4.9E-05	−2.6	8.2E-03
ENSDARG00000091211	adh8a	alcohol dehydrogenase 8a	−4.3	5.9E-03	−4.2	4.6E-03
ENSDARG00000098315	cyp1a	cytochrome P450, family 1, subfamily A	−4.1	1.1E-10	−4.1	4.0E-03
ENSDARG00000101423	cyp2y3	cytochrome P450, family 2, subfamily Y, polypeptide 3	−1.5	1.8E-02	−1.5	4.7E-02
ENSDARG00000103295	cyp3a65	cytochrome P450, family 3, subfamily A, polypeptide 65	−2.7	3.3E-06	−2.7	4.5E-02
ENSDARG00000039832	zgc:173961	zgc:173961	−2.6	1.4E-05	−2.6	1.0E-02
ENSDARG00000090228	gstal	glutathione S-transferase	−2.7	1.1E-05	−2.7	8.7E-03
ENSDARG00000017388	gstt1b	glutathione S-transferase theta 1b	−2.8	1.9E-03	−2.8	8.4E-03

Pathway analysis in Cxcr4b-deficient neutrophils. Genes selected with DESeq (p<0.05) and edgeR (p<0.05) analyses in RStudio (from 21621 to 615 genes) were converted to the human orthologues using g:PROFILER and uploaded in DAVID Bioinformatics.

Resources 6.7 for pathway analysis. Up-regulation of genes involved in focal adhesion/ECMReceptor interaction and axon guidance was identified, whereas down-regulation of genes in the metabolism of xenobiotic by P450 was found. Additional analysis was performed using DESeq2 paired (Table 2). The same pathways were identified with DESeq/edgeR (Table 1) and DESeq2 paired (Table 2) and the genes listed in Table 2 were in addition to genes described in Table 1. Enriched pathways indicate alteration in motility, as shown by the analysis performed with DESeq and edgeR and reveal members of the MAPK signaling to be differentially expressed.

**Table 2:** Enriched pathways in *cxcr4b* ^−/−^ neutrophils (analysis performed with DESeq2 paired).

			DESEq2 paired	
Gene ID	Gene symbol	Gene name	LogFC	p
**Focal adhesion/ECM-receptor interaction**				
ENSDARG00000032639	cd36	CD36 molecule (thrombospondin receptor)	−1.3	8.3E-03
ENSDARG00000012405	col1a1a	collagen, type I, alpha 1a	0.8	2.2E-02
ENSDARG00000061436	col6a2	collagen, type VI, alpha 2	1.0	4.5E-02
ENSDARG00000074316	itga1	integrin, alpha 1	1.1	8.8E-03
ENSDARG00000103056	itga4	integrin alpha 4	0.8	2.6E-02
ENSDARG00000020785	lama4	laminin, alpha 4	1.1	7.1E-03
ENSDARG00000093572	lamc3	laminin, gamma 3	1.5	5.2E-03
ENSDARG00000060711	sv2bb	synaptic vesicle glycoprotein 2Bb	1.7	3.4E-03
ENSDARG00000008867	rap1b	RAP1B, member of RAS oncogene family	−0.9	2.1E-02
ENSDARG00000007825	map2k1	mitogen-activated protein kinase kinase 1	−1.1	2.1E-02
ENSDARG00000098578	pdgfab	platelet-derived growth factor alpha polypeptide b	−1.0	2.1E-02
**Cardiac muscle contraction**				
ENSDARG00000007739	atp1a1a.2	ATPase, Na+/K+ transporting, alpha 1a polypeptide	−1.6	5.8E-05
ENSDARG00000018259	atp1a3a	ATPase, Na+/K+ transporting, alpha 3a polypeptide	0.9	9.6E-03
ENSDARG00000076833	atp1b1b	ATPase, Na+/K+ transporting, beta 1b polypeptide	−1.5	4.0E-04
ENSDARG00000063905	mt-co1	cytochrome c oxidase I, mitochondrial	−0.7	9.3E-03
ENSDARG00000063908	mt-co2	cytochrome c oxidase II, mitochondrial	−0.6	4.1E-02
ENSDARG00000063911	mt-atp6	ATP synthase 6, mitochondrial	−0.7	2.0E-02
ENSDARG00000063912	mt-co3	cytochrome c oxidase III, mitochondrial	−0.7	1.7E-02
ENSDARG00000023886	cacna2d4b	calcium channel, voltage-dependent, alpha 2/delta subunit 4b	1.2	3.2E-02
ENSDARG00000045230	cox6b1	cytochrome c oxidase subunit VIb polypeptide 1	−1.2	4.2E-03
ENSDARG00000038075	cyc1	cytochrome c-1	−0.7	1.9E-02
ENSDARG00000079564	vmhc	ventricular myosin heavy chain	2.1	3.7E-05
**Axon guidance**				
ENSDARG00000044029	efnb3a	ephrin-B3a	1.2	3.2E-02
**MAPK signaling pathway**				
ENSDARG00000008867	rap1b	RAP1B, member of RAS oncogene family	−0.9	2.1E-02
ENSDARG00000035535	rasa1a	RAS p21 protein activator (GTPase activating protein) 1a	0.8	4.5E-02
ENSDARG00000005482	rapgef2	Rap guanine nucleotide exchange factor (GEF) 2	1.0	9.9E-03
ENSDARG00000043241	arrb1	arrestin, beta 1	1.3	3.1E-02
ENSDARG00000023886	cacna2d4b	calcium channel, voltage-dependent, alpha 2/delta subunit 4b	1.2	3.2E-02
ENSDARG00000102474	dusp16	dual specificity phosphatase 16	1.4	9.6E-04
ENSDARG00000061255	dusp3a	dual specificity phosphatase 3a	1.4	1.5E-02
ENSDARG00000009299	dusp8a	dual specificity phosphatase 8a	1.0	3.0E-03
ENSDARG00000092281	FLNB	filamin B	1.3	2.1E-02
ENSDARG00000007825	map2k1	mitogen-activated protein kinase kinase 1	−1.1	2.1E-02
ENSDARG00000001234	map4k2	mitogen-activated protein kinase kinase kinase kinase 2	−1.2	2.9E-02
ENSDARG00000071357	map4k3b	mitogen-activated protein kinase kinase kinase kinase 3b	1.3	3.2E-03
ENSDARG00000070454	pla2g12a	phospholipase A2, group XIIA	−1.4	1.4E-02
ENSDARG00000015662	pla2g12b	phospholipase A2, group XIIB	−1.4	1.4E-02
ENSDARG00000098578	pdgfab	platelet-derived growth factor alpha polypeptide b	−1.0	2.1E-02
ENSDARG00000060551	rps6ka5	ribosomal protein S6 kinase, polypeptide 5	1.5	8.7E-04
ENSDARG00000017494	tgfbr1a	transforming growth factor, beta receptor 1a	1.2	4.1E-02

Pathway analysis in Cxcr4b-deficient neutrophils. Genes selected with DESeq (p < 0.05) and edgeR (p < 0.05) analyses in RStudio (from 21621 to 615 genes) were converted to the human orthologues using g:PROFILER and uploaded in DAVID Bioinformatics Resources 6.7 for pathway analysis. Up-regulation of genes involved in focal adhesion/ECM-Receptor interaction and axon guidance was identified, whereas down-regulation of genes in the metabolism of xenobiotic by P450 was found. Additional analysis was performed using DESeq2 paired (Table 2). The same pathways were identified with DESeq/edgeR (Table 1) and DESeq2 paired (Table 2) and the genes listed in Table 2 were in addition to genes described in Table 1. Enriched pathways indicate alteration in motility, as shown by the analysis performed with DESeq and edgeR and reveal members of the MAPK signaling to be differentially expressed.

### Cxcr4b signaling affects the neutrophilic response to cancer cells during early metastasis formation

**Figure 6:**
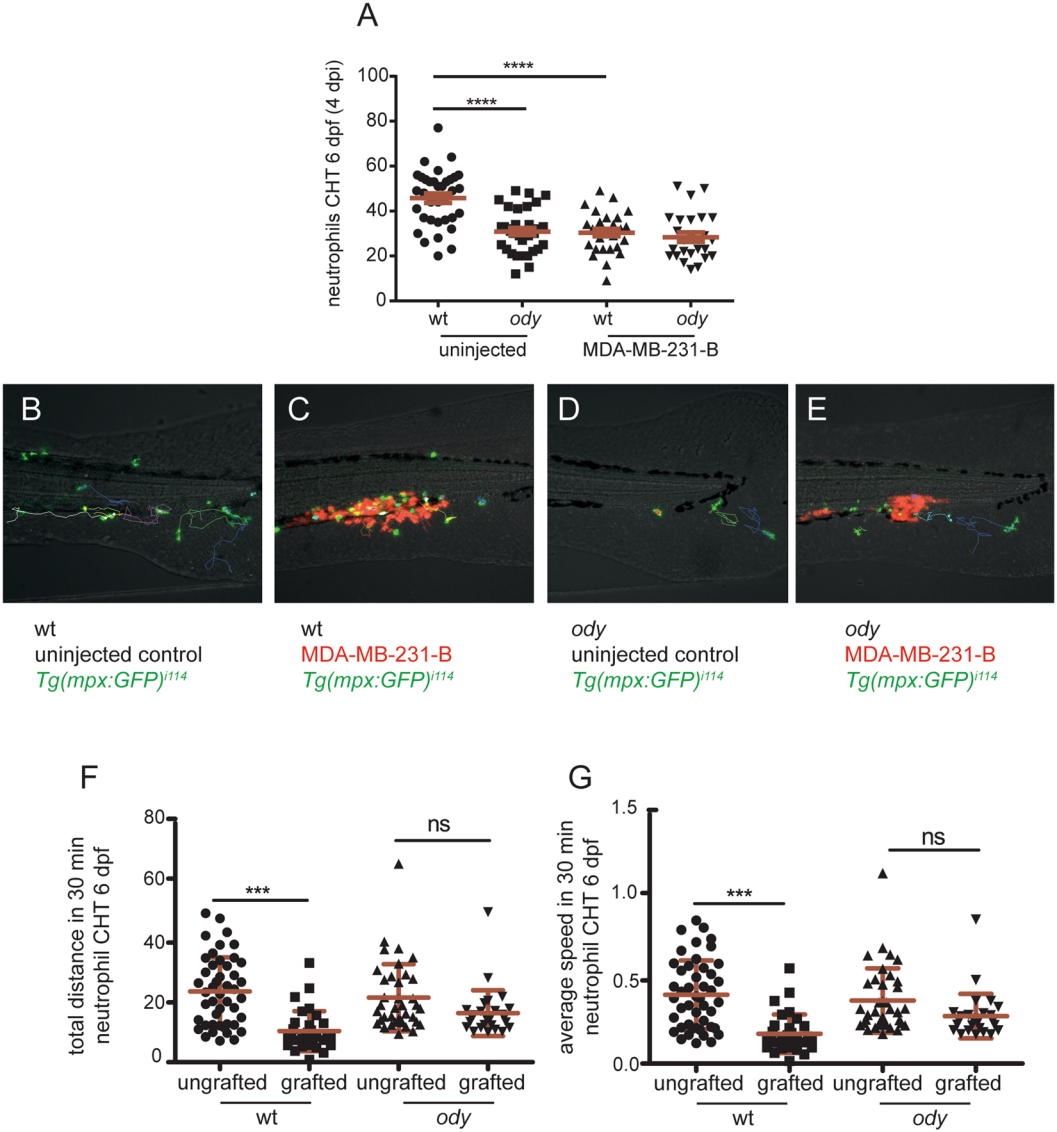
c*xcr4b* loss of function influences neutrophil response to cancer cells initiating early metastases. (**A**) Neutrophil response to metastatic cancer cells was assessed by measuring neutrophil number in the CHT in wt and *ody* larvae at 4 dpi (6 dpf). In control conditions, neutrophils left the CHT when tumor cells were present, whereas they failed to respond, remaining in the CHT, in *ody* mutants (**B**). Kruskal-Wallis, with Dunn *post hoc* test **** p < 0.0001 (number of uninjected embryos is the same as in graph in Fig. 4F; number of engrafted embryos is wt: n = 29 and *ody*: n = 25). Images were acquired using Leica MZ16FA fluorescent microscope coupled to a DFC420C camera. Scale bars: 50 µm. (**B**,**C**) Neutrophil tracking in 6 dpf larvae showed stationary behavior of neutrophils in the presence of tumor cells in wt siblings compared to uninjected controls. (**D**,**E**) Neutrophils maintained the same migratory behavior in *ody* mutants, in presence of MDA-MB-231-B and in uninjected larvae. (**F**,**G**) Neutrophil motility was quantified for 30 minutes, measuring total distance and average speed of each neutrophil in the CHT region. Data are mean ± SD (uninjected wt: n = 46 tracks from 7 larvae; MDA-MB-231-B wt: n = 32 tracks from 5 larvae; uninjected *ody*: n = 37 tracks from 7 larvae; MDA-MB-231-B *ody*: n = 27 tracks from 5 larvae). One-way ANOVA, with Bonferroni *post-hoc* test.

Considering the involvement of Cxcr4b signaling in driving neutrophil motility and development in tumor-naive conditions, next we investigated the ability of neutrophils to respond to cancer cells in *ody* mutants. Generally, emergency hematopoiesis is initiated upon systemic infections and neutrophils leave the bone marrow in response to damage and danger signals, during inflammation and infection^47–50^. Emergency hematopoiesis, dependents on Gcsf-Gcsfr signaling, has also been shown to occur in zebrafish larvae, resulting in expansion of HSPCs and mobilization of neutrophils from the CHT in response to lipopolysaccharide (LPS) injection^51^ or bacterial infection^52^. Hence, the number of neutrophils in the CHT was quantified 3–6 hours after MDA-MB-231-B tumor cells were inoculated into the blood circulation of embryos at 2 dpf. We found that the acute response of neutrophils to tumor cell engraftment was characterized by a decreased number of neutrophils in the CHT in the wt siblings and *od*y embryos, compared to uninjected control groups (Fig. S2A,B). These results suggest that, at 2 dpf, the mobilization of neutrophils from the CHT in response to tumor engraftment is independent from Cxcr4b. As tumor early metastatic events in the CHT region were primarily affected in *ody* mutants at 4 dpi and the CHT colonization by HSPCs is known to occur at 2 dpf, neutrophil response to cancer cells was also assessed at 4 dpi (6 dpf). Like in 2 dpf embryos, we also observed a reduction of neutrophil number in the CHT of tumor-engrafted wt siblings at 6 dpf, compared to the uninjected controls. In contrast, neutrophil numbers were unchanged in tumor-engrafted *ody* mutants, compared to uninjected *ody* larvae (Fig. 6A). Therefore, Cxcr4b signaling is required for the mobilization of neutrophils from the CHT as well tumor-invasive phenotype at 6 dpf.

To further support the evidence that neutrophils display a different response towards cancer cells when Cxcr4b signaling is not functional, we quantified neutrophil motility in the metastatic region at 4 dpi (6 dpf). Neutrophils displayed a motility pattern characterized by lower speed and diminished average distance, in presence of MDA-MB-231-B in the wt siblings, compared to the uninjected controls (Fig. 6B,C,F,G). On the other hand, no differences in neutrophil speed and travelled distance were detected in *ody* larvae implanted with MDA-MB-231-B compared to engrafted wt siblings (Fig. 6D–G). In conclusion, Cxcr4b signaling impairment affects neutrophil response to cancer cells initiating early metastatic events.

## Discussion

Chemokines are key mediators of directional cell migration and the CXCR4-CXCL12 chemokine axis is well known to display major roles in tumor progression, guiding tumor cell homing to CXCL12 expressing organs^53^. Consequently, targeting the CXCR4 receptor expressed by cancer cells is a pharmacological approach that is currently explored in the clinic to limit tumor spreading and metastases^54^. At the same time, it is important to consider the effect of CXCR4 signaling on the tumor microenvironment, especially in view of the antagonizing or supportive functions that myeloid cells are known to have on tumor progression^55^. We previously showed that the zebrafish xenograft model is a powerful tool to study tumor-microenvironment interactions as CXCR4-based interspecies cross talk takes place^12^ and genetic and chemical inactivation of CXCR4 receptor on the engrafted human cancer cells block metastatic onset in zebrafish xenograft model. Moreover, the role of neutrophils in preparing the metastatic niche has been previously described by our group^30^. We found that the non-pathological migration correlate with tumor cell invasion in the caudal hematopoietic tissue (CHT), functionally analogous to the fetal liver in mammalian embryo development. Hence, we hypothesized the involvement of CXCR4 signaling in controlling neutrophil motility and immune-tumor cell interactions involved in the initiation of early metastatic events and micrometastasis formation. First, we found that in zebrafish larvae neutrophils express high levels of *cxcr4b*, the homolog of human *CXCR4* and paralog of zebrafish *cxcr4a*. Then, we used a *cxcr4b* homozygote mutant zebrafish (also known as *odysseus* or *ody*) and showed that engrafted human tumor cells failed to form micrometastases in the CHT region. Therefore, myeloid cell impairment or a non-functional Cxcr4b signaling led to experimental tumor micrometastasis inhibition.

Investigating a potential role of the host Cxcr4b signaling in the formation of early metastasis by affecting immune cell motility was the next approach. We found a downregulation in *mmp9* mRNA levels in *ody* and a reduction in neutrophil motility in tumor-naïve *cxcr4b* deficient zebrafish embryos. These findings link with our previous work on the role of neutrophil physiological migration in tumor invasion in the tail fin^30^. It has been reported that in addition to its function as a protease, mmp9 plays a role as a chemoattractant. Mmp9 chemotactic properties work in synergy with CXCL12^21^. Therefore, inhibition of CXCR4 signaling could lead to impaired neutrophil motility and ability to respond to tumour cells also as a result of altered mmp9-driven chemotaxis. We next investigated whether Cxcr4b signaling affects neutrophil development. In mammals, CXCR4 and CXCR2 chemokine signaling axes regulate hematopoietic stem cell (HSC) retention in and mobilization from the bone marrow, respectively^27,29^. CXCR4 chemical inhibition upon AMD3100 treatment results in mobilized HSCs^56^. Furthermore, patients affected by WHIM syndrome, characterized by neutropenia and enhanced susceptibility to infection, bear a CXCR4-gain-of-function mutation that causes neutrophil retention in hematopoietic sites, in response to cognate ligand CXCL12, highly expressed in the bone marrow^57^. These findings have been confirmed in a zebrafish model of WHIM syndrome, where neutrophils expressing constitutively active Cxcr4b were retained in the CHT and mobilized only upon *cxcl12a* knock down^37^. We found that the number of neutrophils in the CHT in *ody* mutants was higher than in the wt siblings at 2 dpf. Because the overall neutrophil number was not affected by the *cxcr4b* mutation, a higher number of neutrophils in the CHT surprisingly suggested enhanced neutrophil retention. These findings show that receptor stimulation by cognate ligand is needed to activate cell motility, despite of chemotaxis towards Cxcl12. Retention and reduced motility of neutrophils with impaired Cxcr4b signaling at 2 and 3 dpf, respectively, support the hypothesis that neutrophil physiological behavior plays an important role in cancer micrometastasis formation at early stages.

Next we investigated if neutrophils played an important role in preparing the metastatic niche in later stages of tumor micrometastasis formation, when tumor cell invasion has taken place. Therefore, neutrophil number was counted in 6 dpf zebrafish larvae and, in contrast to the observations at 2 dpf, a reduction in neutrophils localized in the CHT was found in *ody* mutants, with tendency towards a reduced overall number in a whole larva. We hypothesize that the dichotomy in neutrophil numbers is linked to the role of CXCR4 signaling during hematopoiesis. Using the zebrafish embryo model, it has recently been shown that HSPCs colonize the hematopoietic tissue, interacting with mesenchymal cells and inducing modification in the perivascular niche. In the same study, the mesenchymal cells express *cxcl12a*, whereas *cxcr4b* expression is mainly found in the CHT region and treatment with the CXCR4 antagonist AMD3100 reduced the number of *runx*^+^ hematopoietic progenitors^40^. In line with their findings, we propose that the reduced number of neutrophils in the CHT of 6 dpf *ody* larvae relates to the reduced number of HSPCs and suggest that further investigations should be carried on to confirm this hypothesis. Importantly, a lower number of neutrophils in the CHT in 6 dpf *ody* mutants might result in a reduced niche modification due to a lower number of paths traced into the collagen by neutrophils themselves. On the other end, the increased number of neutrophils in earlier stages suggests the potential role of Cxcr4b in the primitive wave of hematopoiesis.

In agreement with our findings, Cxcr4b signature in tumor-naïve zebrafish neutrophils confirmed Cxcr4b role in cell motility and adhesion. Upregulation of the integrins, as well as increased interaction with the ECM and alteration of the cytoskeleton reorganization were found in Cxcr4b deficient neutrophils. Members of the Roundabout signaling pathway were also differentially expressed. Roundabout signaling is associated with axon guidance mechanisms and its role in cancer metastasis has been reported^58^. Importantly, *Slit1b*, found up-regulated in *ody*, functions as a repellent molecule that interferes with leukocyte chemotaxis^59^ and specifically blocks the ability of circulating neutrophils to migrate directionally^60^. Moreover, we propose *netrin1b* as a candidate gene that links neutrophil ability to provide trophic signals to cancer cells. *NETRIN-1* has been reported to reduce neutrophil infiltration in ischemic acute kidney injury by inhibiting COX-2 and PGE2 production^46^. PGE2 has been identified as the trophic signal that sustains neoplastic transformation in a transgenic zebrafish cancer model^15^.

**Figure 7:**
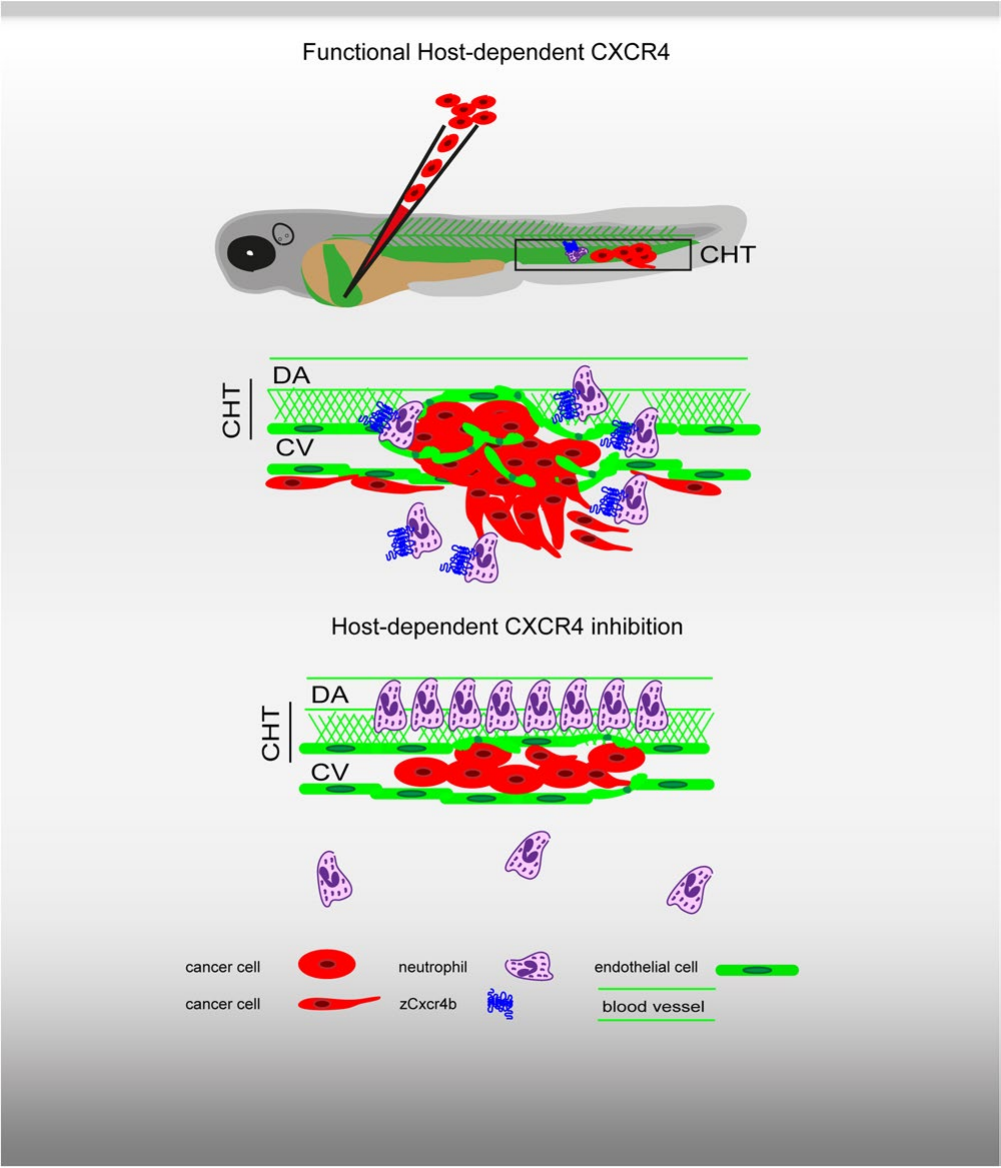
Role of host-dependent CXCR4 signaling in experimental metastasis formation in the zebrafish xenograft model. Inoculation of human tumor cells into the blood circulation of zebrafish embryos results in experimental metastasis formation, characterized by tumor cell aggregates in the blood vessels, and extravasation and invasion in the surrounding tissue, in the region of the caudal hematopoietic tissue (CHT). During early metastatic events, endothelium alteration takes place and neutrophils localize in the surrounding of the tumor cells. The CHT is a vascular plexus in the tail fin between the DA and the CV and is a hematopoietic site. Upon disruption of the host CXCR4 (Cxcr4b) signaling, cancer cells are unable to initiate early metastatic events. Neutrophils are preferentially retained in the CHT and fail to accumulate at tumor metastatic sites.

After investigating the role of Cxcr4b in physiological neutrophil development and motility, we unraveled neutrophil behavior in presence of engrafted tumor cells, able to initiate early metastatic events. An acute response to engrafted cancer cells into the blood circulation of 2 dpf zebrafish embryos resulted in no alteration in Cxc4b-deficient neutrophils. To assess neutrophil acute response in tumor-engrafted larvae, the number of *mpx*^+^ cells was counted in the CHT of zebrafish embryos few hours after tumor cell inoculation. As neutrophil number decreased in the CHT of engrafted wt or *ody* embryos compared to uninjected larvae, we propose that neutrophils mount an acute response upon tumor inoculation by leaving the CHT in line with previous observations of demand-driven granulopoiesis upon bacterial infection^52^ and that this response occurs in a Cxcr4b independent manner. On the other hand, an altered response was found at later stages. In 6 dpf (4 dpi) zebrafish larvae, tumor cells have formed a secondary tumor mass and initiated local tissue invasion. In response, wt siblings diminished the number of neutrophils in the CHT, increasing their mobilization. Mobilized neutrophils were found to migrate and in the surrounding of metastasizing cancer cells and to slow down and to interact with human malignant cells. In contrast, *cxcr4b* deficient neutrophils remained in the CHT and failed to localize in the surrounding of tailfin tumor micrometastases, suggesting a possibly diminished inflammatory response (Fig. 7).

In conclusion, we demonstrate that CXCR4 signaling plays a major role in neutrophilic innate immune response to early metastatic events and contributes to the establishment of tumor micrometastases. The development of CXCR4-targeted therapies directed to the tumor microenvironment is therefore essential.

## Materials and Methods

### Zebrafish husbandry

Zebrafish lines were kept in compliance with the local animal welfare regulations and European directives. The study was approved by the local animal welfare committee (DEC) of the University of Leiden (license number: 10612, protocol 14227). Zebrafish adults were maintained according to standard protocols (zfin.org), in a 10/14-hour dark/light cycle. Embryos were maintained at 28 °C in Egg water (60 µg/ml Ocean salt in distilled water), containing 0.003% PTU (1-phenyl-2-thiourea) to block pigmentation.

### Zebrafish lines

Zebrafish reporter lines used in this study were *Tg(mpx:GFP)*^*i114*^ ^34^ and *Tg(Kdrl:EGFP)*^*s843*^ ^61^. The *cxcr4b*^*t26035*^ line^36^ was outcrossed with each reporter line mentioned above. Homozygote *cxcr4b*^−/−^ mutant embryos (*odysseus or ody*) were distinguished from wild type *cxcr4b*^+/+^ and heterozygote *cxcr4b*^+/−^ siblings by phenotype (incomplete lateral line deposition) and genotype identification. Genotyping of adult fish by KASP assay was performed using the following primers A1 (reverse) 5′-TGACGGTGGTCTTCAGTGCCTT-3′, A2 (reverse) 5′-TGACGGTGGTCTTCAGTGCCTA-3′ and C1 (forward) 5′-CAAGAACTCCAAGGGTCAGACTCTA-3′ and confirmed by sequencing using previously described primers^62^.

### Cell culture

Breast MDA-MB-231-B dsRed^32^ (kindly provided by P. ten Dijke and Y. Drabsch, LUMC, Leiden, The Netherlands), MDA-MB-157 mCherry (ATCC^®^) and prostate PC3-M-Pro4-Luc2 (mCherry or tdTomato) (kindly provided by G. van der Pluijm, LUMC, Leiden, The Netherlands) cancer cell lines were cultured in DMEM medium complemented with 10% fetal calf serum (FCS), at 37 °C in a humidified atmosphere with 5% CO_2_. Cell lines were regularly tested for mycoplasma with Universal Mycoplasma Detection kit (30–1012 k, ATCC).

### Pu.1 knock down

Pu.1 (Spi1b, 1 mM) and standard control morpholino injections (0.1 mM) were performed to deplete neutrophils and macrophages as previously described^30^. Morpholino efficiency was assessed by counting number of Mpx^+^ neutrophils in the *Tg(mpx:GFP)*^*i114*^ zebrafish line.

### Xenograft experiments

Tumor cells were inoculated in the blood circulation of 2 day post fertilization (dpf) zebrafish embryos as previously described^33^.

### Tumor burden

Zebrafish embryos engrafted with fluorescently labelled tumor cells were screened for correct engraftment 5–6 hours after inoculation into the blood circulation at 2 dpf, using a Leica MZ16FA fluorescent microscope coupled to a DFC420C camera. Larvae were positioned on a Petri dish with 1.5% agarose coating and tumor burden was quantified at 4 dpi, acquiring monographs of the metastatic site, in the CHT region. LAS AF Lite software was used to overlay the GFP and dsRed channels and snapshots were analyzed in Image-Pro Analyzer 7.0 (Media Cybernetics). For each larva tumor burden was calculated based on number of objects multiplied by mean area and mean intensity, generated with a macro designed by H. de Bont (Toxicology, LACDR, Leiden University) and previously used to quantify tumor migration and proliferation^63,64^.

### Neutrophil number and motility

Neutrophil number was quantified by manual counting, using a Leica MZ16FA fluorescent microscope. Neutrophil basal motility was assessed using a Leica TCS SPE confocal microscope with a HC APO 20x DRY objective (0.7 N.A.). 3 dpf larvae were mounted on a 1% low melting point agarose layer, containing tricaine and covering the glass surface of a Will-Co Dish^®^ (Pelco^®^, Ted Pella, Inc). Egg water containing anesthetic was added on top of each larva. Timelapse was performed for 30 minutes, with 1 minute interval between frames. Maximum projections were generated, tail movements were corrected using Stack Reg plugin and neutrophil tracking was performed using the Manual Tracking plugin in ImageJ-Fiji^65^. Neutrophil motility in response to metastatic cancer cells was quantified with a Nikon A1 confocal laser scanning microscope (Tokyo, Japan) using the 488 and 561 laser lines with 20 × (NA 0.75) lens. Images were acquired every minute during timelapse. Videos were analyzed using NIS-Elements AR and tracking performed for the first 30 frames in ImageJ, with Manual Tracking plugin.

### RNA isolation and real-time PCR

Expression levels of *mmp9* were quantified in 6 dpf *cxcr4b*^*t26035*^ *Tg(Kdrl:EGFP)*^*s843*^ larvae. RNA was isolated using TRIZOL extraction method, according to the manufacturer’s instruction from a pool of zebrafish larvae (10 < n < 15). DNase treatment was performed using RQ1 RNase free-DNase (M6101 Promega). 1 μg input RNA was used for cDNA synthesis (i-Script™ cDNA synthesis kit, Bio-Rad). Expression levels were measured by real-time PCR (iQ™ SYBR® Green Supermix, Bio-Rad), using the Chromo4™ Four-Color Real-Time PCR system. Relative fold changes of gene expression were calculated using the ΔΔCt method. The following primers were used: *mmp9* forward 5′CATTAAAGATGCCCTGATGTATCCC -3′ and mmp9 reverse 5′-AGTGGTGGTCCGTGGTTGAG-3′^66^. Peptidylprolyl isomerase A-like (*ppiaI*) was used as housekeeping gene (forward 5′-ACACTGAAACACGGAGGCAAAG -3′ and reverse RV 5′- CATCCACAACCTTCCCGAACAC-3′).

### *cxcr4b* transcriptomic signature in neutrophils: from larval dissociation to RNA sequencing analysis

Zebrafish line *cxcr4b*^*t26035*^ *Tg(mpx:GFP)*^*i114*^ was used to isolate neutrophils from 6 dpf larvae. After harvesting, eggs were kept in Petri dishes (n ≤ 100) at 28.5 °C to allow synchronized embryo development. Triplicates of GFP positive embryos (100–150 per replicate) were selected for dissociation, performed according to^67^. Dissociation with 0.4 mg/ml collagenase/DPBS (Liberase TL, Roche, #05401020001) was alternatively used. Larvae were transferred directly from Egg water to collagenase solution. Dissociation was obtained mechanically with pipetting and 2 incubation steps at 28.5 °C for 10 min. 10% FCS was added and sample preparation was continued as described in^67^. Sorting was performed with a BD FACSAria™ III Cell Sorter (BD Biosciences, San Jose, CA, USA) with the BD FACSDiva software (version 6.1.3) and gates defined using GFP negative larvae. After sorting, samples were stored in QIAzol at −80 °C. RNA isolation was performed using miRNeasy Mini kit (# 217004 Qiagen). On-column DNase digestion was performed, using RNase-Free DNase Set (# 79254 Qiagen). Agilent Bioanalyzer 2100, RNA 6000 Pico kit (Agilent, Santa Clara) was used to assess RNA quality. cDNA synthesis and amplification were performed with SMARTer® Ultra™ Low Input RNA Kit for Sequencing - v3 (Clontech) and cDNA quality validated, using Agilent 2100 BioAnalyzer and the High Sensitivity DNA Chip from Agilent’s High Sensitivity DNA Kit (#5067-4626, Agilent). cDNA shearing, library preparation and validation, and Illumina sequencing (HiSeq2000) were performed as described in^67^ by ZF-SCREENS (Leiden, The Netherland). Reads (18.684.327 is an average of 12 samples) were mapped to Ensembl transcripts (GRCz10.80) and statistical analysis based on negative binomial distribution performed in R Studio, using DESEq, DESEq2 paired and EdgeR packages, available at Bioconductor.org. Pathway analysis was performed using DAVID Bioinformatics Resources 6.7. Identification of *cxcr4a* and *cxcr4b* expression levels in neutrophils by RNA sequencing shown in Fig. 2 was performed as described in^67^.

### Statistics

Statistical analysis was performed using GraphPad Prism (versions 5.0 and 6.0). Un-paired t-test was used in datasets of two groups and Welch’s correction applied when group variances were significantly different (p < 0.05). One-way ANOVA with Bonferroni *post hoc* test was used in datasets of three or more groups (continuous variable) and Kruskal-Wallis with Dunn’s *post hoc* test was used to estimate significant difference in the case of counts (discrete variable).

## Supplementary information

Supplementary Figures and Figure Legends

## Data Availability

Data generated or analysed during this study are included in this published article (and its Supplementary Information files). The datasets generated and/or analysed during the current study are available from the corresponding author on reasonable request.
