# Supplementary material for: CXCR4 signaling regulates metastatic onset by controlling neutrophil motility and response to malignant cells
Source: Sci Rep. 2019 Feb 20;9:2399. doi: 10.1038/s41598-019-38643-2 (PMC6382824; doi:10.1038/s41598-019-38643-2)
Supplement: Supplementary file 1 — Supplementary Figures and Figure Legends [file 41598_2019_38643_MOESM1_ESM.docx]

**CXCR4 signaling regulates metastatic onset by controlling neutrophil motility and response to malignant cells**

Tulotta C., Stefanescu C., Chen Q., Torraca V., Meijer A.H. and Snaar Jagalska B.E.

**Supplementary Figures**

**
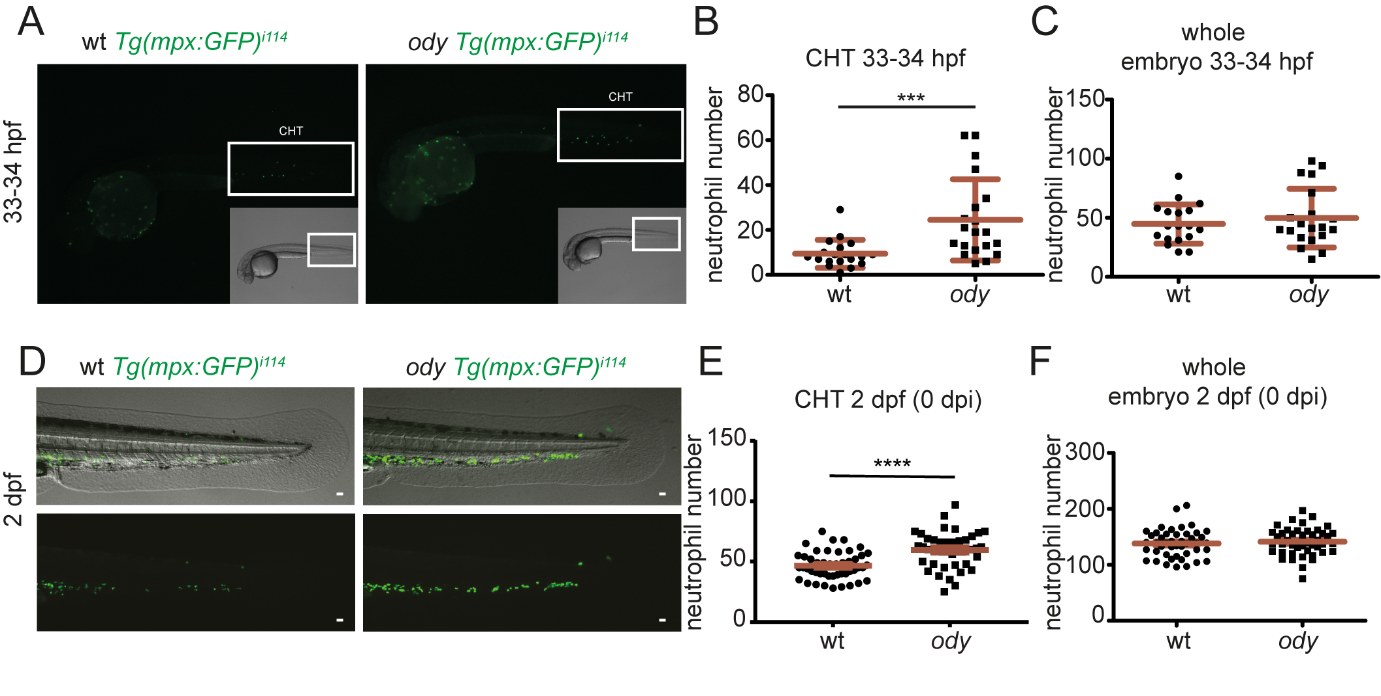
**

**Figure S1. Cxcr4b and hematopoiesis**

(A-C) Neutrophil number was counted in wt and *ody* siblings at 33-34 hpf, when neutrophil development is independent from HSPC colonization of the CHT, indicated by the box (A). (B) A significantly higher number of neutrophils was present in the CHT region in *ody* embryos, compared to wt siblings, while the total neutrophil number was found unchanged in *cxcr4b^+/+^* and *cxcr4b^-/-^* (C). Data are mean±SD. Mann-Whitney test ***p<0.0004. In (B) and (C) n=19 (wt) and n=20 (*ody*). (D, E) Neutrophils were counted in the CHT in wt and *ody* mutants at 2 dpf and a significant increment was found in *ody* embryos (31% increase) (D, top panel is a bright field image of bottom panel showing GFP^+^ neutrophils; E, data quantification). (E) Un-paired t-test, with Welch’s correction Mann-Whitney test ****p<0.0001. Data are mean ± SEM of two independent experiments (wt: n=47, *ody*: n=45). (F) Total neutrophil number was counted in wt and *ody* mutants at 2 dpf and no difference was found. In (F) wt: n=45, *ody*: n=45.


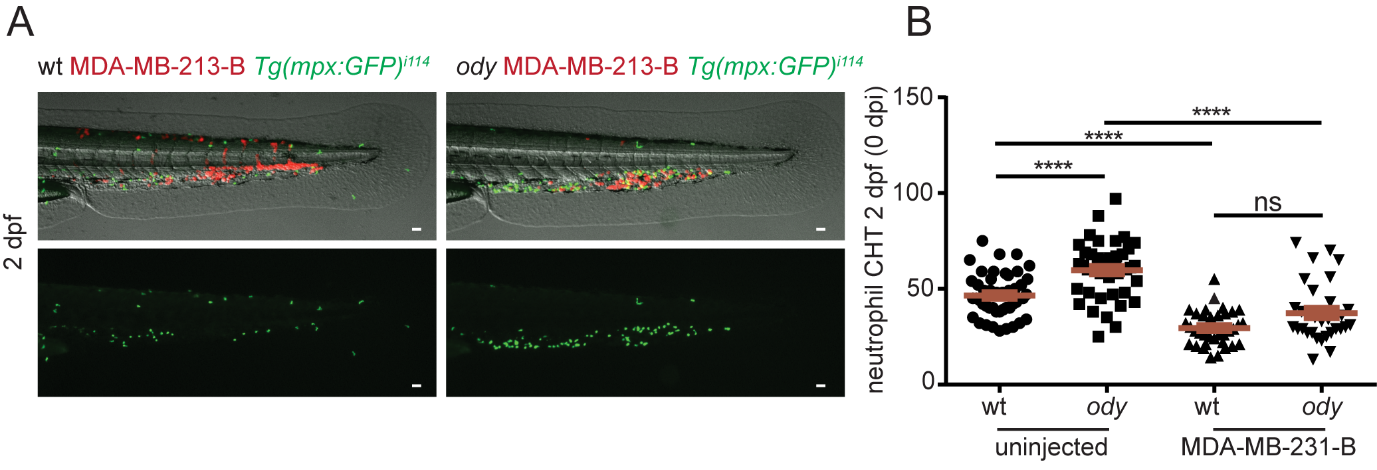


**Figure S2. Neutrophil mobilization in response to malignant cells is independent from cxcr4b during emergency hematopoiesis.** (A, B) Neutrophil response to cancer cells ~3-6 hours after engraftment in the blood circulation of 2 dpf zebrafish embryos, wt or *ody* mutant, in a *Tg(mpx:GFP)^i114^* background. (A, top panel is an overlay of bright field, DsRed^+^ and GFP^+^ channels and corresponds to the bottom panel where only GFP^+^ neutrophils are shown). (B) Neutrophils exited the CHT in response to tumor cells, both in wt and *ody* embryos. Kruskal-Wallis, with Dunn *post hoc* test **** p<0.0001, ns p>0.05 (number of uninjected embryos is the same as in graph in Figure S1F; number of engrafted embryos is wt: n=35 and Ody: n=32). Images were acquired using Leica MZ16FA fluorescent microscope coupled to a DFC420C camera. Scale bars: 50 µm.
